# A novel method for estimating distributions of body mass index

**DOI:** 10.1186/s12963-016-0076-2

**Published:** 2016-03-12

**Authors:** Marie Ng, Patrick Liu, Blake Thomson, Christopher J. L. Murray

**Affiliations:** 1 Institute for Health Metrics and Evaluation, Seattle, USA; 2 IBM Watson Health, San Jose, USA; 3 University of Cambridge, Cambridge, UK

**Keywords:** BMI distribution, Overweight, Obesity, L-BFGS-B optimization, Beta distribution

## Abstract

**Background:**

Understanding trends in the distribution of body mass index (BMI) is a critical aspect of monitoring the global overweight and obesity epidemic. Conventional population health metrics often only focus on estimating and reporting the mean BMI and the prevalence of overweight and obesity, which do not fully characterize the distribution of BMI. In this study, we propose a novel method which allows for the estimation of the entire distribution.

**Methods:**

The proposed method utilizes the optimization algorithm, L-BFGS-B, to derive the distribution of BMI from three commonly available population health statistics: mean BMI, prevalence of overweight, and prevalence of obesity. We conducted a series of simulations to examine the properties, accuracy, and robustness of the method. We then illustrated the practical application of the method by applying it to the 2011–2012 US National Health and Nutrition Examination Survey (NHANES).

**Results:**

Our method performed satisfactorily across various simulation scenarios yielding empirical (estimated) distributions which aligned closely with the true distributions. Application of the method to the NHANES data also showed a high level of consistency between the empirical and true distributions. In situations where there were considerable outliers, the method was less satisfactory at capturing the extreme values. Nevertheless, it remained accurate at estimating the central tendency and quintiles.

**Conclusion:**

The proposed method offers a tool that can efficiently estimate the entire distribution of BMI. The ability to track the distributions of BMI will improve our capacity to capture changes in the severity of overweight and obesity and enable us to better monitor the epidemic.

## Introduction

Overweight and obesity are growing health problems worldwide. In 2013, nearly one third of the world’s population was either overweight or obese [1]. Concern regarding the rising disease burden associated with obesity has become nearly universal, and widespread calls have been made for more consistent and accurate monitoring in all populations [2].

Conventional strategies for monitoring population-level overweight and obesity rely on obtaining point estimates, including mean body mass index (BMI) or prevalence of overweight (BMI ≥ 25) and obesity (BMI ≥ 30) [3, 4]. Mean and prevalence are succinct metrics which provide useful insight into distinct aspects of a population’s distribution of BMI. In addition, these measures are easily interpreted by the general public. However, to rigorously monitor the rapidly evolving obesity epidemic, simply observing measures of mean and prevalence is not adequate. Specifically, as the proportion of overweight and obesity increases, the distribution of BMI will become skewed. This, in turn, affects the ability of mean to accurately reflect the central tendency of the distribution [5–7]. If the goal is simply to obtain a more accurate estimate of central tendency, it may be sufficient to replace mean by median. However, as the epidemic intensifies, there is a growing interest in understanding the shift in the BMI distribution and in tracking changes across subclasses of obesity which include class I (BMI: 30–34.9), class II (BMI: 35–39.9), and class III obesity (BMI ≥ 40) [8]. Furthermore, understanding population distribution of BMI is critical to estimating the associated disease burden. To calculate the population attributable fraction of diseases related to high BMI, for instance, one would need to have an accurate measure of exposure represented by population BMI distribution [9]. Therefore, there is a practical need to look beyond measures of mean and prevalence and to monitor the distribution of BMI as a whole.

Monitoring the population distribution of BMI is a challenging task. Existing national surveillance systems do not always include a sample size sufficient for precise approximations of BMI distributions by subpopulation, such as by sex and age [10]. A direct solution would be to increase sample sizes of a survey. However, given the need for regular and timely monitoring, increasing sample sizes will be costly and may not be sustainable in the long run. It would, therefore, be highly desirable to develop a strategy that can effectively use available point estimates from surveys to infer the underlying distribution.

In this study, we propose a novel method that utilizes an optimization algorithm to approximate the distribution of BMI using the three commonly available population-level metrics: mean BMI, prevalence of overweight, and prevalence of obesity. The paper is organized as follows: We first provide a brief description of the proposed method. We then describe the simulation experiment used to validate the method and present the results. To illustrate the utility of the method, we apply it to the 2011–2012 US National Health and Nutrition Examination Survey (NHANES) and compare our estimate with the true distribution of BMI. We conclude by discussing the potential extension, limitations, and implications of the method.

## Methods

### Rationale

The characteristics of a continuous distribution are defined by its probability density function (pdf). Depending on the distribution, the parameters involved in the pdf vary. For instance, a normal distribution has a pdf defined by a measure of central tendency (μ) and a measure of dispersion (σ^2^) parameters. On the other hand, a beta distribution has a pdf defined by two shape parameters, namely α and β. Although estimates of these parameters are not always immediately available, they can be easily derived from any two pieces of known statistical information.

In the case of BMI, three statistics which are commonly available from existing surveys are mean BMI, prevalence of overweight, and prevalence of obesity. They respectively provide information on central tendency and specific quintiles. Based on this information and assumptions about the potential family of distributions, parameters can be obtained analytically. For example, if a normal distribution is assumed, μ can be immediately inferred from the sample mean. σ^2^ (assuming that sample variance information is not immediately available) can be calculated based on the mean and quintiles using inverse z scores. Suppose prevalence of obesity is 0.025; if BMI is normally distributed, the corresponding z-value would be 1.96. Using the standard z-score calculation formula, $z=\frac{X-\mu }{\sigma }$, with *z* = 1.96, *X* = 30, *μ* being the sample mean, σ^2^ can be calculated. Once μ and σ^2^ are defined, the shape of the distribution is fully realized.

The issue with assuming a normal distribution, however, is that as the epidemic shifts, the shape of the BMI distribution will begin to skew. In other words, to accurately capture this shift, the distribution assumed needs to be flexible enough to represent both symmetric and asymmetric patterns. Some of the potential distribution candidates include log normal, and the gamma, beta, and inverse Gaussian distributions. To determine which would best approximate the distribution of actual data, we briefly examined national survey data from the most recent years from six countries with measured height and weight for men and women. The skewness of BMI distributions in these survey data ranged from 0.68 to 1.43, and the kurtosis ranged from 3.94 to 8.96 (see Table 1). While log normal and the gamma distributions offer fit to a variety of unimodal distributions, some of the shapes generated by these two distributions have extreme skewness and kurtosis which are not suitable for the situation at hand. Moreover, for both log normal and the gamma distributions, skewness and kurtosis are defined by a single parameter, which limits their flexibility in capturing distribution of particular shapes. Inverse Gaussian distribution offers reasonable fit to skewed data with varying levels of kurtosis. However, in situations where the data distribution is relatively symmetric, Inverse Gaussian may not be flexible enough to capture them. In contrast, with certain constraints imposed (see next section), the beta distribution offers a variety of symmetric light-tailed and asymmetric heavy-tailed distributions which possess skewness and kurtosis within the observed range. The proposed method capitalizes on the flexibility of the beta distribution to estimate the entire BMI distribution based on information about the mean and quintiles. Further detail is provided in subsequent sections.

**Table 1:** Skewness and kurtosis of BMI distributions from six countries

ISO3	Survey	Year	Sex	Skewness	Kurtosis
UGA	DHS	2011	Male	0.82	4.87
UGA	DHS	2011	Female	1.37	8.96
IND	DHS	2005	Male	1.26	7.17
IND	DHS	2005	Female	1.43	7.29
SAU	Saudi Arabia HIS	2013	Male	0.77	4.22
SAU	Saudi Arabia HIS	2013	Female	0.68	3.94
DOM	DHS	2013	Male	1.10	5.72
DOM	DHS	2013	Female	0.90	4.37
GBR	Health Survey for England	2011	Male	0.94	5.56
GBR	Health Survey for England	2011	Female	1.02	4.47
USA	NHANES	2011	Male	1.13	5.59
USA	NHANES	2011	Female	1.27	5.84

### Estimation of BMI distribution

To estimate the distribution of BMI, we assume that:

*BMI* = *C*_1_*u* + *C*_2_ *C*_1_ > 0, *C*_2_ ≥ 10

where *C*_1_ is a positive scaling constant and *C*_2_ is a shifting constant. Note that a constraint of greater than or equal to 10 was imposed on *C*_2_. Because the lower limit of a population BMI distribution rarely falls below 10, imposing this constraint enhances the accuracy of the optimization results. *u* is a random variable following the beta distribution with values ranging from zero to one.

*u* ∼ *Beta* (*α*, *β*), *α* > 1, *β* > 1

where *α* and *β* are the shape parameters. When *α* > 1, *β* > 1, and *α* = *β*, the beta distribution is unimodal and symmetric. When *α* > 1, *β* > 1, and *α* < *β* , the distribution is unimodal and positively skewed. In contrast with other distributions such as log normal and gamma, a beta distribution is relatively light-tailed and provides more stable estimation at the tails of the distribution.

Estimates of *α*, *β*, *C*_1_, and *C*_2_ are obtained by minimizing the following function:

$$ D=\left|\right|\boldsymbol{s} - \boldsymbol{t}\left|\right| = \sqrt{\left(\boldsymbol{s} - \overset{.}{\boldsymbol{t}}\right)\cdotp \left(\boldsymbol{s} - \boldsymbol{t}\right)} $$

where *D* refers to the Euclidean distance, which is the shortest distance between two points, in this case two vectors *s* and *t. s* is a vector consisting of the observed mean BMI (${\overline{X}}_{bmi}$) and prevalence of overweight and obesity (${\overline{p}}_{bmi\ge 25},{\overline{p}}_{bmi\ge 30}$); *t* is a vector consisting of the predicted mean BMI (${\tilde{X}}_{bmi}$) and prevalence (${\tilde{p}}_{bmi\ge 25},{\tilde{p}}_{bmi\ge 30}$) for a given set of *α*, *β*, *C*_1_, and *C*_2_. The large-scale bound-constrained optimization algorithm (L-BFGS-B) was used for the optimization [11]. Other optimization algorithms, including conjugate gradient, Nelder-Mead, and Broyden-Fletcher-Foldfarb-Shannon algorithms were considered. However, L-BFGS-B was chosen as it provided a much more efficient optimization process and more stable results.

### Simulation

**Fig. 1:**
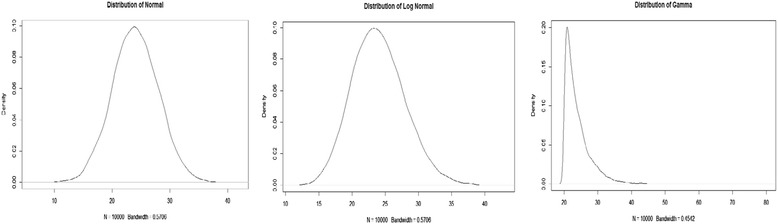
Distributions used for simulation. The normal distribution (left) represents a symmetric light-tailed distribution; the log normal distribution (center) represents a slightly skewed and light-tailed distribution; the gamma distribution (right) represents a skewed and heavy-tailed distribution

A series of simulations were carried out to examine the performance of the method. Data were simulated from three distributions representing different levels of skewness and kurtosis similar to those observed in the survey data. The first set of data were simulated from a normal distribution. The second and third sets of data were simulated from a log normal and a gamma distribution, respectively. The normal distribution represents a symmetric light-tailed distribution; the log normal distribution represents a slightly skewed and light-tailed distribution; the gamma distribution represents a skewed and heavy-tailed distribution. The intention for simulating from log normal and the gamma distribution is to test the robustness of the method in handling extreme scenarios. If the method performs well under these extreme circumstances, it offers confidence for general use. For consistency, we centered and scaled all three distributions to ensure that they had a mean of 24 and a standard deviation of four. Table 2 summarizes the characteristics of the distributions. Graphical displays of each distribution are shown in Fig. 1.

**Table 2:** Descriptive statistics of the distributions considered for simulation

	Normal	Log normal^a^	Gamma^a^
Parameters	μ = 24 (mean) σ = 4 (standard deviation)	Log(μ) = 0 (log mean) Log(σ) = 0.1 (log standard deviation)	κ = 1 (shape) θ = 2 (scale)
Mean	24		
Standard deviation	4		
Skewness	0.007	0.31	1.95
Kurtosis	2.95	3.12	8.47

^a^Data generated from log normal and the gamma distributions were scaled to have mean of 24 and standard deviation of four

A random sample of 500 observations were drawn from each of the three distributions. The sample mean and prevalence of overweight and obesity were calculated. Based on these three statistics, we applied the proposed method to approximate the distribution of BMI. The process was repeated 1000 times for each of the distributions.

To determine how well the empirical distribution estimated from the method approximates the true distribution, we evaluated the biases and mean squared errors in four key statistics: mean, standard deviation, the prevalence of overweight, and the prevalence of obesity. Specifically, for mean, the bias is calculated by the difference between the expected mean BMI derived from the empirical distribution across the 1000 simulations and the true mean of 24:

$$ Bia{s}_{\overline{X}} = E\left({\overline{X}}_{bmi}\right)-{\mu}_{bmi} $$

For standard deviation and prevalence of overweight and obesity, the biases are calculated in a similar manner, as follows:

$$ Bia{s}_{sd} = E\left({S}_{bmi}\right)-{\sigma}_{bmi} $$

$$ Bia{s_{\widehat{p}}}_{{}_{\ge 25}} = E\left({\widehat{p}}_{\ge 25}\right)-{p}_{\ge 25} $$

$$ Bia{s}_{{\widehat{p}}_{\ge 30}} = E\left({\widehat{p}}_{\ge 30}\right)-{p}_{\ge 30} $$

where *S*_*bmi*_, ${\widehat{p}}_{\ge 25}$, and ${\widehat{p}}_{\ge 30}$ are the standard deviation, prevalence of overweight, and prevalence of obesity estimated respectively from the empirical distribution for a simulated data set. On the other hand, mean squared errors are calculated as follows:

$$ MS{E}_{\overline{X}} = E\left({\left({\overline{X}}_{bmi}-{\mu}_{bmi}\right)}^2\right) $$

$$ MS{E}_{sd} = E\left({\left({S}_{bmi}-{\sigma}_{bmi}\right)}^2\right) $$

$$ ME{S}_{{\widehat{p}}_{\ge 25}}=E\left({\left({\widehat{p}}_{\ge 25}-{p}_{\ge 25}\right)}^2\right) $$

$$ MS{E}_{{\widehat{p}}_{\ge 30}}=E\left({\left({\widehat{p}}_{\ge 30}-{p}_{\ge 30}\right)}^2\right) $$

In addition to calculating bias and mean squared errors, the Kolmogrov-Smirnov test was performed to examine how well the predicted distributions matched the actual distributions of the sample. We computed the proportion of the time in which the test falsely rejected the null hypothesis that the empirical and true distribution are equal with *α* = 0.05.

### Applied example

Further validation was performed using data from the 2011–2012 NHANES. Specifically, based on mean and prevalence information by age and sex, we estimated the distributions of BMI for males and females for each 10-year age group from 20 to 70+ years old. The empirical distributions were compared against the distribution of actual data using the Kolmogrov-Smirnov test.

All analyses were conducted in R 3.0.1.

## Results and discussion

### Simulation

Overall, the proposed methods performed well across all scenarios (see Table 3). The biases in the key distribution parameters were minimal. Specifically, the estimated means were consistently similar to the true mean of 24, with biases ranging from −0.036 to −0.006. The biases in standard deviation estimates were slightly larger, ranging from −0.121 to −0.026. The prevalence of overweight and obesity derived from the empirical distribution was equal to the true values (bias of zero).

**Table 3:** Biases and mean squared errors (in parentheses) in estimated parameters, and Kolmogrov-Smirnov test results

		Normal	Log normal	Gamma
Bias (MSE)	$\overline{X}$	−0.006	−0.010	−0.036
		(0.058)	(0.062)	(0.068)
	*SD*	−0.121	−0.070	−0.026
		(0.035)	(0.035)	(0.146)
	${\widehat{p}}_{\ge 25}$	0.001	0.001	0.016
		(0.001)	(0)	(0.001)
	${\widehat{p}}_{\ge 30}$	0	−0.002	0.003
		(0)	(0)	(0)
Kolmogrov-Smirnov test false rejection rate		2.5 %	2.3 %	24.9 %

**Fig. 2:**
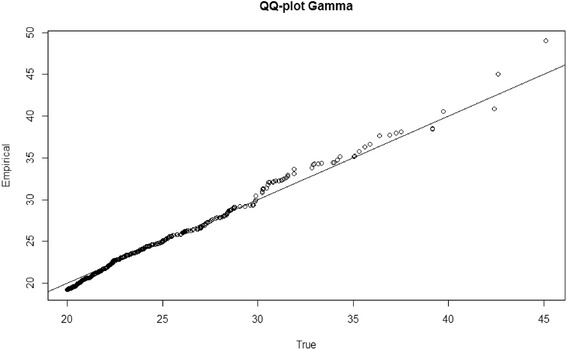
An example of a QQ-plot indicating the discrepancy between the empirical and true (the gamma) distributions. Deviation from the 45° line represents a lack of alignment between the two distributions. Major discrepancies existed at the right tail where the method failed to precisely capture the extreme values

Despite the consistency in the point estimates, the Kolmogrov-Smirnov test indicated that, in some cases, certain aspects of the true distribution were not captured by the empirical distribution. When the true BMI distribution was normal or log normal, the method performed reasonably well. Only 2.5 % and 2.3 % of the 1000 empirical distributions, respectively, exhibited a statistically significant deviation from the true distribution. These rates are considered to be desirable given the α-level of 5 % [12]. However, as the distribution becomes more skewed and heavy-tailed, the discrepancy between the empirical distribution and the true distribution increases. When the true BMI distribution was gamma, approximately 24.9 % of the 1000 empirical distributions exhibited a statistically significant deviation from the true distribution. Further investigation was carried out to identify potential causes of the discrepancy. Figure 2 shows a QQ-plot illustrating the quintile at which discrepancies existed between the empirical and true distributions, with the true distribution being the gamma distribution. As the plot suggests, the discrepancies were mainly restricted to the right tail where the method failed to precisely capture the extreme values. In other words, the presence of outliers and the sparse data at extreme ends pose challenges to the accuracy of the method. Nevertheless, it is worth emphasizing that both the log normal and the gamma distributions represent a relatively high level of kurtosis, meaning that these distributions tend to have heavy tails with extreme values. Moreover, considering the fact that the underlying distribution assumed by the method is distinct from the simulated distributions, the method’s capability in approximating the central tendency and quintiles of these alternative distributions is considered robust.

### Applied example

**Fig. 3:**
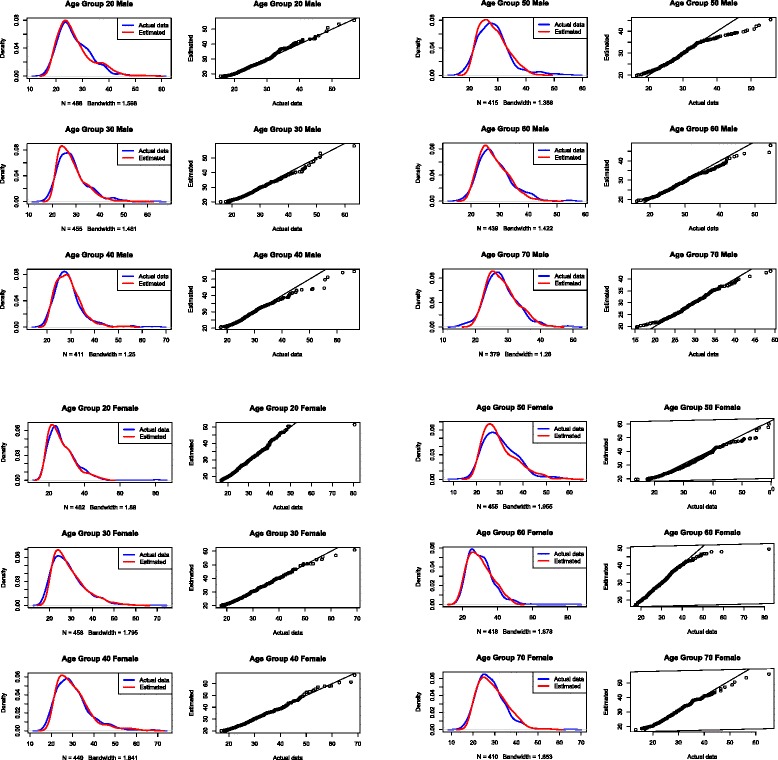
Comparison between the empirical distributions and the true data distribution and corresponding QQ-plots. Overlapping lines in the density plots indicate the empirical distributions were reasonably accurate at approximating the distributions of actual data. The QQ-plots similarly suggests minor deviations at the tail of the distributions for some age groups

We applied the proposed method to data from the 2011–2012 NHANES. Using only the mean BMI, prevalence of overweight, and prevalence of obesity for each sex and 10-year age group (from ages 20-70+), we approximated the distributions for each of these subgroups and compared them against the distributions of the actual data. Figure 3 shows the differences between the empirical and the true data distributions. As indicated by the overlapping lines in the density plots, the empirical distributions were reasonably accurate at approximating the distributions of actual data. The QQ-plots similarly suggest that our method accurately approximated the distribution of true data with minor deviations at the tail of the distributions for some age groups, such as males ages 40–49 and 50–59. The Kolmogrov-Smirnov test results (Table 4) indicated that there is no statistically significant difference between the empirical and true distributions.

**Table 4:** Kolmogrov-Smirnov Test results (*p*-values) comparing the empirical distribution to the NHANES data distribution

	Kolmogrov-Smirnov test statistics and p-values	
Age group	Male	Female
20-29	0.043	0.069
	*p = 0.755*	*p = 0.218*
30-39	0.064	0.066
	*p = 0.314*	*p = 0.280*
40-49	0.046	0.056
	*p = 0.772*	*p = 0.490*
50-59	0.048	0.081
	*p = 0.721*	*p = 0.099*
60-69	0.068	0.043
	*p = 0.475*	*p = 0.833*
70+	0.036	0.063
	*p = 0.958*	*p = 0.381*

## Conclusions

In this study, we proposed a novel method to approximate the entire distribution of BMI using three commonly available statistics, namely mean BMI and prevalence of overweight and obesity. We assessed the method using a series of simulations, and the results indicated that the method performed well in approximating distributions with a wide range of skewness and kurtosis. We illustrated the application of the method using data from NHANES, which similarly demonstrated the accuracy of the approach.

A major appeal of the proposed method lies in its use of readily available health statistics. Distributions of BMI can be approximated without the need to collect a large amount of data. Moreover, past BMI distributions can be retrospectively constructed using historical information on mean BMI and prevalence of overweight and obesity. In addition, the current method is robust and can adequately estimate distributions which do not conform with the underlying distribution (beta distribution) assumed by the method. As part of the Global Burden of Disease Study 2013, we applied the proposed method to historical data to reconstruct the BMI distributions by age and sex for 192 countries from 1980 to 2013. Without utilizing the new approach, obtaining precise BMI distributions would have been impossible as in many countries historical individual-level BMI data were unavailable [13].

One of the limitations of this method, however, is the reduction in accuracy when the true distribution contains outliers. Specifically, our method may be inadequate at capturing outliers at the tails of a distribution. This limitation may be due to the assumption of the beta distribution in our approximation strategy. Although the beta distribution offers the flexibility to model a wide variety of distributional shapes, it is relatively weak at handling extreme kurtosis. Alternative distributions such as log normal and the gamma offer better capability at capturing long, heavy-tailed distributions. Nevertheless, the lack of finite upper bounds for these distributions posed challenges in the optimization process, which led to instability in estimation.

Despite this limitation, results from our simulations showed that the prevalence of overweight and obesity estimated from the empirical distributions are unbiased. This implies that, although the method may be limited in identifying the precise BMI value of outliers, it is able to offer an accurate approximation of the *proportion* of extreme values. Additionally, it is worth emphasizing that the design of the method is very flexible. For this simulation, three values were utilized in the optimization function. Additional statistics, such as prevalence of underweight and prevalence of different obesity classes, could be easily incorporated to the method and improve the accuracy of the distribution approximation.

In summary, the algorithm proposed in this paper serves as an efficient method to approximate BMI distributions. In fact, this algorithm can be applied to estimating the distribution of other continuous risk factors such as blood pressure and glucose level and facilitate more accurate assessment of associated disease burden. While the method performed well in various situations, some aspects can be improved. Future studies can explore non-parametric density approximation techniques to expand the flexibility of the method.
